# Methyl 1-bromo-2-naphthoate

**DOI:** 10.1107/S1600536809050466

**Published:** 2009-11-28

**Authors:** Zong-Ling Ru, Guo-Xi Wang

**Affiliations:** a Department of Chemical & Environmental Engineering, Anyang Institute of Technology, Anyang 455000, People’s Republic of China

## Abstract

In the mol­ecular structure of the title compound, C_12_H_9_BrO_2_, the methoxy­carbonyl group is twisted by a dihedral angle of 29.8 (3)°with respect to the naphthalene ring system. An overlapped arrangement is observed between parallel naphthalene ring systems of adjacent mol­ecules, and the face-to-face distance of 3.590 (9) Å suggests there is π–π stacking in the crystal structure.

## Related literature

For the chemistry of naphthoate derivatives, see: Ballabh *et al.* (2005 ▶); Imai *et al.* (2006 ▶).
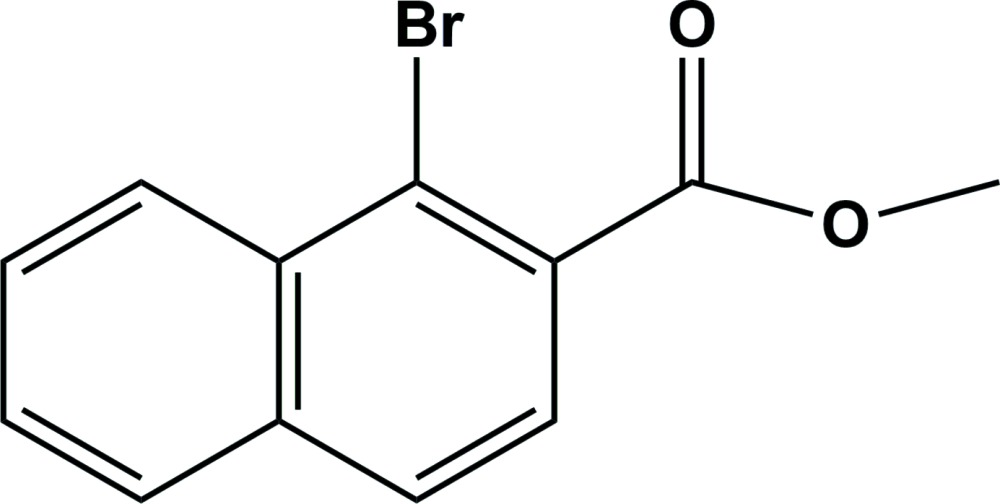


## Experimental

### 

#### Crystal data

C_12_H_9_BrO_2_
*M* *_r_* = 265.10
Monoclinic, 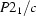

*a* = 9.3614 (19) Å
*b* = 9.3014 (19) Å
*c* = 12.069 (2) Å
β = 93.66 (3)°
*V* = 1048.7 (4) Å^3^
*Z* = 4
Mo *K*α radiation
μ = 3.89 mm^−1^
*T* = 298 K
0.4 × 0.35 × 0.2 mm

#### Data collection

Rigaku Mercury2 diffractometer
Absorption correction: multi-scan (*CrystalClear*; Rigaku, 2005 ▶) *T*_min_ = 0.881, *T*_max_ = 0.940
10520 measured reflections
2400 independent reflections
1751 reflections with *I* > 2σ(*I*)
*R*_int_ = 0.086

#### Refinement

*R*[*F* ^2^ > 2σ(*F* ^2^)] = 0.052
*wR*(*F* ^2^) = 0.127
*S* = 1.06
2400 reflections
137 parameters
H-atom parameters constrained
Δρ_max_ = 0.41 e Å^−3^
Δρ_min_ = −0.51 e Å^−3^

### 

Data collection: *CrystalClear* (Rigaku, 2005 ▶); cell refinement: *CrystalClear*; data reduction: *CrystalClear*; program(s) used to solve structure: *SHELXTL* (Sheldrick, 2008 ▶); program(s) used to refine structure: *SHELXTL*; molecular graphics: *SHELXTL* software used to prepare material for publication: *SHELXTL*.

## Supplementary Material

Crystal structure: contains datablocks I, global. DOI: 10.1107/S1600536809050466/xu2693sup1.cif

Structure factors: contains datablocks I. DOI: 10.1107/S1600536809050466/xu2693Isup2.hkl

Additional supplementary materials:  crystallographic information; 3D view; checkCIF report

## Figures and Tables

**Fig. 1.**
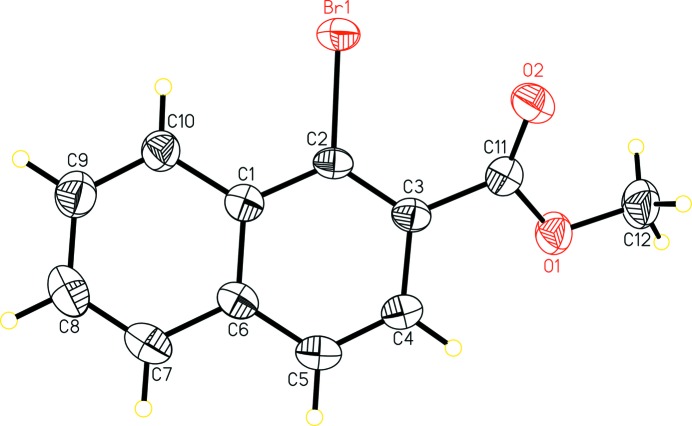
A view of the title compound with the atomic numbering scheme. Displacement ellipsoids were drawn at the 30% probability level.

## supplementary crystallographic information

### Comment

Naphthoate derivatives are an important class of chemical raw materials, which have found wide range of applications in catalytic reaction, coordination chemistry as ligand, dye industry, and which are also used in medicine as drugs, such as adapalene. Recently, a series of naphthoate compounds have been reported (Ballabh *et al.*, 2005; Imai *et al.*, 2006). As an extension of these work on the structural characterization, we report here the crystal structure of the title compound methyl 1-bromo-2-naphthoate.

The crystal data show that in the title compound (Fig.1), the two benzene rings are essentially coplanar and only twisted from each other by a dihedral angle of 1.11 (2)°. All the bond length are within the normal range. An overlapped arrangement is observed between parallel naphthalene ring systems of adjacent molecules, and the face-to-face distance of 3.590 (9) Å suggests there is π–π stacking in the crystal structure.

### Experimental

The purchased 1-bromo-2-naphthoate (3 mmol, 795 mg) was dissolved in chloroform (20 ml) and evaporated in the air affording colorless block crystals of this compound suitable for X-ray analysis were obtained.

### Refinement

All H atoms bonded to C atoms were fixed geometrically and treated as riding with C–H = 0.93 Å(aromatic), C–H =0.96 Å(methyl), with *U*_iso_(H) = 1.2Ueq(aromatic) and *U*_iso_(H) = 1.5Ueq(methyl).

### Crystal data

**Table:**

C_12_H_9_BrO_2_	*F*(000) = 528
*M**_r_* = 265.10	*D*_x_ = 1.679 Mg m^−^^3^
Monoclinic, *P*2_1_/*c*	Mo *K*α radiation, λ = 0.71073 Å
Hall symbol: -P 2ybc	Cell parameters from 1751 reflections
*a* = 9.3614 (19) Å	θ = 3.1–27.5°
*b* = 9.3014 (19) Å	µ = 3.89 mm^−^^1^
*c* = 12.069 (2) Å	*T* = 298 K
β = 93.66 (3)°	Block, colourless
*V* = 1048.7 (4) Å^3^	0.4 × 0.35 × 0.2 mm
*Z* = 4	

### Data collection

**Table:**

Rigaku Mercury2 diffractometer	2400 independent reflections
Radiation source: fine-focus sealed tube	1751 reflections with *I* > 2σ(*I*)
graphite	*R*_int_ = 0.086
Detector resolution: 13.6612 pixels mm^-1^	θ_max_ = 27.5°, θ_min_ = 3.1°
ω scans	*h* = −12→12
Absorption correction: multi-scan (*CrystalClear*; Rigaku, 2005)	*k* = −12→12
*T*_min_ = 0.881, *T*_max_ = 0.940	*l* = −15→15
10520 measured reflections	

### Refinement

**Table:**

Refinement on *F*^2^	Primary atom site location: structure-invariant direct methods
Least-squares matrix: full	Secondary atom site location: difference Fourier map
*R*[*F*^2^ > 2σ(*F*^2^)] = 0.052	Hydrogen site location: inferred from neighbouring sites
*wR*(*F*^2^) = 0.127	H-atom parameters constrained
*S* = 1.06	*w* = 1/[σ^2^(*F*_o_^2^) + (0.053*P*)^2^] where *P* = (*F*_o_^2^ + 2*F*_c_^2^)/3
2400 reflections	(Δ/σ)_max_ < 0.001
137 parameters	Δρ_max_ = 0.41 e Å^−^^3^
0 restraints	Δρ_min_ = −0.50 e Å^−^^3^

### Special details

**Table:**

Geometry. All e.s.d.'s (except the e.s.d. in the dihedral angle between two l.s. planes) are estimated using the full covariance matrix. The cell e.s.d.'s are taken into account individually in the estimation of e.s.d.'s in distances, angles and torsion angles; correlations between e.s.d.'s in cell parameters are only used when they are defined by crystal symmetry. An approximate (isotropic) treatment of cell e.s.d.'s is used for estimating e.s.d.'s involving l.s. planes.
Refinement. Refinement of *F*^2^ against ALL reflections. The weighted *R*-factor *wR* and goodness of fit *S* are based on *F*^2^, conventional *R*-factors *R* are based on *F*, with *F* set to zero for negative *F*^2^. The threshold expression of *F*^2^ > σ(*F*^2^) is used only for calculating *R*-factors(gt) *etc*. and is not relevant to the choice of reflections for refinement. *R*-factors based on *F*^2^ are statistically about twice as large as those based on *F*, and *R*- factors based on ALL data will be even larger.

### Fractional atomic coordinates and isotropic or equivalent isotropic displacement parameters (Å^2^)

**Table:**

	*x*	*y*	*z*	*U*_iso_*/*U*_eq_	
Br1	0.29147 (5)	0.80840 (4)	0.47073 (3)	0.0664 (2)	
C2	0.4234 (4)	0.6858 (3)	0.4033 (3)	0.0450 (8)	
C1	0.5663 (4)	0.6855 (3)	0.4501 (3)	0.0485 (9)	
C4	0.4815 (4)	0.5034 (4)	0.2735 (3)	0.0553 (9)	
H4	0.4533	0.4410	0.2159	0.066*	
C3	0.3790 (4)	0.5986 (3)	0.3159 (3)	0.0471 (8)	
C5	0.6197 (5)	0.5021 (4)	0.3156 (3)	0.0608 (10)	
H5	0.6849	0.4405	0.2852	0.073*	
C6	0.6655 (4)	0.5916 (4)	0.4039 (3)	0.0512 (9)	
C10	0.6150 (5)	0.7727 (4)	0.5411 (3)	0.0596 (10)	
H10	0.5517	0.8347	0.5733	0.072*	
C9	0.7542 (5)	0.7662 (5)	0.5817 (4)	0.0715 (12)	
H9	0.7842	0.8239	0.6417	0.086*	
C8	0.8523 (5)	0.6753 (5)	0.5355 (5)	0.0774 (14)	
H8	0.9468	0.6730	0.5642	0.093*	
C12	0.0587 (5)	0.4584 (5)	0.1631 (4)	0.0812 (13)	
H12A	0.0394	0.3588	0.1475	0.122*	
H12B	0.0531	0.5120	0.0950	0.122*	
H12C	−0.0107	0.4948	0.2112	0.122*	
C7	0.8094 (5)	0.5901 (5)	0.4484 (4)	0.0699 (12)	
H7	0.8754	0.5298	0.4174	0.084*	
C11	0.2313 (4)	0.5993 (4)	0.2617 (3)	0.0552 (9)	
O1	0.1996 (3)	0.4727 (3)	0.2164 (2)	0.0678 (7)	
O2	0.1531 (4)	0.7005 (3)	0.2538 (3)	0.0835 (10)	

### Atomic displacement parameters (Å^2^)

**Table:**

	*U*^11^	*U*^22^	*U*^33^	*U*^12^	*U*^13^	*U*^23^
Br1	0.0647 (3)	0.0697 (3)	0.0652 (3)	0.02213 (19)	0.0071 (2)	−0.01421 (18)
C2	0.050 (2)	0.0373 (17)	0.0492 (19)	0.0072 (14)	0.0111 (17)	0.0042 (14)
C1	0.052 (2)	0.0429 (19)	0.051 (2)	0.0000 (16)	0.0059 (18)	0.0077 (15)
C4	0.062 (3)	0.049 (2)	0.056 (2)	0.0044 (18)	0.0101 (19)	−0.0056 (17)
C3	0.050 (2)	0.0426 (18)	0.0495 (19)	0.0051 (15)	0.0090 (17)	0.0060 (15)
C5	0.062 (3)	0.055 (2)	0.067 (2)	0.012 (2)	0.016 (2)	−0.0015 (19)
C6	0.048 (2)	0.047 (2)	0.059 (2)	0.0036 (16)	0.0093 (18)	0.0099 (16)
C10	0.058 (3)	0.052 (2)	0.068 (3)	−0.0023 (18)	−0.003 (2)	−0.0026 (18)
C9	0.073 (3)	0.065 (3)	0.075 (3)	−0.004 (2)	−0.006 (3)	−0.002 (2)
C8	0.054 (3)	0.079 (3)	0.098 (4)	−0.004 (2)	−0.010 (3)	0.015 (3)
C12	0.073 (3)	0.085 (3)	0.083 (3)	−0.014 (2)	−0.016 (3)	−0.009 (2)
C7	0.053 (3)	0.070 (3)	0.087 (3)	0.012 (2)	0.011 (2)	0.010 (2)
C11	0.058 (2)	0.055 (2)	0.053 (2)	−0.0032 (19)	0.0025 (18)	−0.0014 (17)
O1	0.0675 (19)	0.0576 (16)	0.0759 (18)	−0.0024 (14)	−0.0136 (15)	−0.0074 (14)
O2	0.067 (2)	0.0716 (19)	0.109 (2)	0.0204 (15)	−0.0172 (19)	−0.0198 (16)

### Geometric parameters (Å, °)

**Table:**

Br1—C2	1.901 (3)	C10—H10	0.9300
C2—C3	1.374 (5)	C9—C8	1.390 (7)
C2—C1	1.418 (5)	C9—H9	0.9300
C1—C6	1.415 (5)	C8—C7	1.357 (6)
C1—C10	1.417 (5)	C8—H8	0.9300
C4—C5	1.360 (5)	C12—O1	1.436 (5)
C4—C3	1.425 (5)	C12—H12A	0.9600
C4—H4	0.9300	C12—H12B	0.9600
C3—C11	1.491 (5)	C12—H12C	0.9600
C5—C6	1.398 (5)	C7—H7	0.9300
C5—H5	0.9300	C11—O2	1.193 (4)
C6—C7	1.418 (5)	C11—O1	1.324 (4)
C10—C9	1.364 (6)		
			
C3—C2—C1	122.4 (3)	C1—C10—H10	119.8
C3—C2—Br1	120.7 (3)	C10—C9—C8	121.5 (4)
C1—C2—Br1	116.9 (2)	C10—C9—H9	119.2
C6—C1—C10	118.1 (4)	C8—C9—H9	119.2
C6—C1—C2	118.1 (3)	C7—C8—C9	119.7 (4)
C10—C1—C2	123.8 (3)	C7—C8—H8	120.1
C5—C4—C3	121.1 (3)	C9—C8—H8	120.1
C5—C4—H4	119.4	O1—C12—H12A	109.5
C3—C4—H4	119.4	O1—C12—H12B	109.5
C2—C3—C4	117.8 (3)	H12A—C12—H12B	109.5
C2—C3—C11	124.1 (3)	O1—C12—H12C	109.5
C4—C3—C11	118.1 (3)	H12A—C12—H12C	109.5
C4—C5—C6	121.2 (4)	H12B—C12—H12C	109.5
C4—C5—H5	119.4	C8—C7—C6	121.0 (4)
C6—C5—H5	119.4	C8—C7—H7	119.5
C5—C6—C1	119.4 (4)	C6—C7—H7	119.5
C5—C6—C7	121.4 (4)	O2—C11—O1	123.2 (4)
C1—C6—C7	119.3 (4)	O2—C11—C3	125.9 (3)
C9—C10—C1	120.4 (4)	O1—C11—C3	110.8 (3)
C9—C10—H10	119.8	C11—O1—C12	116.3 (3)
			
C3—C2—C1—C6	0.2 (5)	C10—C1—C6—C7	−1.2 (5)
Br1—C2—C1—C6	177.9 (2)	C2—C1—C6—C7	179.4 (3)
C3—C2—C1—C10	−179.1 (3)	C6—C1—C10—C9	0.5 (5)
Br1—C2—C1—C10	−1.5 (4)	C2—C1—C10—C9	179.9 (4)
C1—C2—C3—C4	1.2 (5)	C1—C10—C9—C8	0.3 (6)
Br1—C2—C3—C4	−176.4 (2)	C10—C9—C8—C7	−0.4 (7)
C1—C2—C3—C11	−177.7 (3)	C9—C8—C7—C6	−0.3 (7)
Br1—C2—C3—C11	4.7 (4)	C5—C6—C7—C8	−178.7 (4)
C5—C4—C3—C2	−2.1 (5)	C1—C6—C7—C8	1.1 (6)
C5—C4—C3—C11	176.9 (4)	C2—C3—C11—O2	30.4 (6)
C3—C4—C5—C6	1.6 (6)	C4—C3—C11—O2	−148.5 (4)
C4—C5—C6—C1	−0.1 (5)	C2—C3—C11—O1	−153.4 (3)
C4—C5—C6—C7	179.7 (4)	C4—C3—C11—O1	27.7 (4)
C10—C1—C6—C5	178.6 (3)	O2—C11—O1—C12	−4.8 (6)
C2—C1—C6—C5	−0.8 (5)	C3—C11—O1—C12	178.9 (3)
